# Editorial: Multi-omics and molecular biology studies on abiotic stress in crops

**DOI:** 10.3389/fgene.2025.1607710

**Published:** 2025-07-03

**Authors:** Xiaobo Luo, Georgia Ntatsi, Rong Zhou, Mintao Sun

**Affiliations:** ^1^ State Key Laboratory of Vegetable Biobreeding, Institute of Vegetables and Flowers, Chinese Academy of Agricultural Sciences, Beijing, China; ^2^ Guizhou Institute of Biotechnology, Guizhou Academy of Agricultural Sciences, Guiyang, China; ^3^ Laboratory of Vegetable Production, Department of Crop Science, Agricultural University of Athens, Athens, Greece; ^4^ Sanya Institute of Nanjing Agricultural University, Nanjing Agricultural University, Nanjing, Jiangsu, China

**Keywords:** transcriptomics, metabolomics, gene family abundance, plant stress, molecular mechanisms

## Introduction

Plants face a multitude of biotic and abiotic stresses that threaten their growth, development, and productivity. Biotic stresses, such as fungal infections, and abiotic stresses, including drought, salinity, and temperature extremes, activate complex molecular networks involving transcriptional reprogramming, metabolic adjustments, and signaling cascades. Recent advancements in omics technologies have enabled comprehensive exploration of these mechanisms across diverse plant species. This review synthesizes findings from six studies on lily (*Lilium* spp.), licorice (*Glycyrrhiza uralensis*), potato (*Solanum tuberosum*), rice (*Oryza sativa*), soybean (*Glycine max*), and pigeonpea (*Cajanus cajan*), focusing on their molecular strategies to combat stress. By integrating transcriptomic, metabolomic, and gene family analyses, we highlight conserved pathways, species-specific adaptations, and future directions for crop improvement.

## Transcriptional and metabolic reprogramming in lily bulb rot resistance

In this Research Topic Chang et al. showed that *Fusarium oxysporum*-induced lily bulb rot triggers dynamic transcriptomic shifts, with 3,922, 7,595, and 6,590 DEGs at early (LYBH2), mid- (LYBH3), and late-stage (LYBH4) infection, respectively. In this study, key upregulated TFs—WRKY (regulating lignin via SA/JA signaling; Rushton et al., 1996; Deng et al., 2023) and AP2/ERF (modulating SA/ET/JA pathways; Ma et al., 2017b) were found to drive phenylpropanoid-derived antimicrobials.

The metabolomic analysis identified stage-specific flavonoids: Kaempferol-3-O-rutinoside-7-O-rhamnoside (LYBH2, antibacterial; Ma et al., 2017b), quercetin-3-O-glucoside (LYBH3, antiviral; Wei et al., 2021), and lignification enhancers (LYBH4; Ninfali et al., 2020). Despite upregulated lignin genes (PAL, CCoAOMT; Sun et al., 2024), minimal metabolite shifts suggest post-transcriptional regulation.

## Soybean drought response: physiology, transcriptome and metabolome

In the study of Wang et al., drought stress was found to reduce photosynthesis and water use efficiency (WUE), with non-stomatal limitations dominating under severe drought (SD). Rehydration restored WUE in moderate drought (MD) but not severe drought (SD), indicating irreversible damage (Qi et al., 2021). Moreover, the chlorophyll fluorescence parameters (*Fv/Fm*, ΦPSII) mirrored photosynthetic recovery under drought stress.

The transcriptome analysis in this study, revealed that drought stress induced the expression of the *PAO1, 4, 5* and *P5CS* genes to promote the accumulation of spermidine and proline, enhancing soybean drought tolerance. Moreover, the metabolome analysis also identified proline, DL-tryptophan, and phenylalanine as key osmolytes under drought stress. Proline accumulation in MD plants aligned with barley and wheat studies (Chmielewska et al., 2016), while tryptophan derivatives may correlate with antioxidant responses (Rabara et al., 2017). Integrated transcript-metabolite networks highlighted phenylpropanoid and amino acid pathways as critical hubs.

## MAPK signaling in licorice salt stress adaptation

Gao et al., revealed that the MAPK cascade, conserved across eukaryotes, transduces stress signals via phosphorylation (Jagodzik et al., 2018). In *G. uralensis*, 21 GuMAPKs were classified into four subgroups (A–D) based on TEY/TDY activation motifs (López-Bucio et al., 2014). Subgroups A (GuMAPK3/6) and D (GuMAPK16) exhibited colinearity with Arabidopsis and tomato homologs, underscoring evolutionary conservation. Within GuMAPKs, gene duplication, particularly segmental duplication, drove functional diversification, as seen in three homologous pairs (Wang et al., 2021).

Under 200 mM NaCl, *GuMAPK*5, 7, 9, and 16 were upregulated, while *Bacillus subtilis* inoculation further enhanced their expression, indicating microbial priming of salt tolerance. Protein interaction networks linked GuMAPKs to PR1 (pathogenesis-related protein) and RBOHD (ROS-generating NADPH oxidase), bridging biotic and abiotic stress responses (Yamada et al., 2016). At 300 mM NaCl, *GuMAPK16-2* downregulation post-inoculation suggested stress threshold modulation.

## *COLs* gene family in potato tuberization and cold stress

Yin et al., discovered that potato tuberization is regulated by photoperiod-sensitive *StCOL* genes (Abelenda et al., 2016). Phylogenetic analysis classified *StCOLs* into three subfamilies with conserved motifs/structures (2–4 exons) and 10 motifs/6 PTMs affecting protein function. Synteny revealed 13 *StCOLs* share a common ancestor, highlighting evolutionary conservation. Cold-responsive *StCOL2, 3, 9, and 15* contained low-temperature cis-elements. *StCOL9* downregulation post-chilling suggests its role as a negative regulator, akin to *AtCOL1* in Arabidopsis (Mikkelsen and Thomashow, 2009). These genes likely integrate photoperiod and temperature cues to optimize tuberization under stress.

## *ALOGs* gene family in rice development and abiotic stress

Liu et al., explained that the ALOG domain, derived from retroposon recombinases, governs rice reproductive development (Turchetto et al., 2023). Phylogenetic analysis divided 14 *OsG1L* genes into six clades, with *OsG1L1/2/5/6* regulating panicle architecture (Beretta et al., 2023). Collinearity between *OsG1L3/4/5* and *OsG1L7/8* suggested subfunctionalization. Rice *ALOG* promoters are enriched with ABA-responsive ABRE motifs (half with ≥5 ABREs; up to 12 in one member) and drought-linked MBS elements. Most *ALOG* genes are downregulated under ABA/drought, consistent with ABA-insensitive root/seed phenotypes in LSH8 mutants (promoter ABREs, nuclear localization; Zou et al., 2021). Similarly, *OsG1L7* (9 ABREs, nuclear) is suppressed by ABA/drought, suggesting shared roles in ABA signaling. These findings highlight *ALOG* family involvement in ABA-mediated stress responses via promoter cis-elements and transcriptional regulation.

## *BAGs* gene family in pigeonpea and their response in thermotolerance

The study by Alekhya et al. conducted a comprehensive genomic and functional characterization of the BAG gene family in pigeonpea (*C. cajan*), revealing critical insights into their role in heat stress response. Alekhya et al., demonstrated that Pigeonpea’s nine *BAGs* genes (five chromosomes) show lineage-specific evolution via Whole Genome Duplication (WGD). UBL domains in BAG1/2/4 suggest ubiquitin roles, while BAG6’s IQ motif links to calcium signaling. Phylogenetically, five clades (shared with tomato/soybean) reflect exon/intron divergence, with non-conserved structures (as in Arabidopsis, rice, wheat (Doukhanina et al., 2006; Rana et al., 2012; Ge et al., 2016) driving functional diversification.

In heat-tolerant genotype TS3R, *CcBAG4* (interacting with HSP70) was upregulated, suppressing cell death (Doukhanina et al., 2006). Conversely, *CcBAG5/6* showed upregulation in susceptible lines, mirroring tomato *SlBAG9* (homolog of *AtBAG5*) overexpression-induced heat sensitivity (Ding et al., 2022). MiRNA targeting of *CcBAG6* in TS3R suggested post-transcriptional silencing, enhancing thermotolerance.

## Convergent mechanisms and future perspectives

Conserved stress-response mechanisms across species involve transcriptional hubs (WRKY, AP2/ERF, NAC TFs) coordinating stress-specific gene regulation, metabolic pathways (phenylpropanoid/amino acid biosynthesis) producing chemical defenses, and signaling networks (MAPK cascades, BAG-HSP chaperones) linking stress perception to protection. Translational innovations include CRISPR editing (e.g., StCOL9, OsG1L7) for climate resilience, microbiome engineering (*B. subtilis*) priming MAPK pathways, and metabolic engineering (proline/lignin) enhancing drought/fungal resistance. These strategies integrate molecular insights with biotechnology, offering scalable solutions for sustainable crop improvement amid climate challenges.
